# Selenium and Chronic Diseases: A Nutritional Genomics Perspective

**DOI:** 10.3390/nu7053621

**Published:** 2015-05-15

**Authors:** Catherine Méplan

**Affiliations:** 1 School of Biomedical Sciences, Faculty of Medical Sciences, Newcastle University, Newcastle upon Tyne NE2 4HH, UK; 2 Human Nutrition Research Centre, Newcastle University, Newcastle upon Tyne NE2 4HH, UK; E-Mail: Catherine.meplan@ncl.ac.uk; Tel.: +44-191-208-7664; Fax: +44-191-208-8900

**Keywords:** selenoprotein P, glutathione peroxidase, single nucleotide polymorphisms, cancer, nutritional genomics, selenium

## Abstract

Mechanistic data have revealed a key role for selenium (Se) and selenoproteins in biological pathways known to be altered in multifactorial diseases, such as cellular maintenance, response to oxidative stress and correct protein folding. Although epidemiological studies indicate that low Se intake is linked to increased risk for various chronic diseases, supplementation trials have given confusing outcomes, suggesting that additional genetic factors could affect the relationship between Se and health. Genetic data support this hypothesis, as risk for several chronic diseases, in particular cancer, was linked to a number of single nucleotide polymorphisms (SNP) altering Se metabolism, selenoprotein synthesis or activity. Interactions between SNPs in selenoprotein genes, SNPs in related molecular pathways and biomarkers of Se status were found to further modulate the genetic risk carried by the SNPs. Taken together, nutritional genomics approaches uncovered the potential implication of some selenoproteins as well as the influence of complex interactions between genetic variants and Se status in the aetiology of several chronic diseases. This review discusses the results from these genetic associations in the context of selenoprotein functions and epidemiological investigations and emphasises the need to assess in future studies the combined contribution of Se status, environmental stress, and multiple or individual SNPs to disease risk.

## 1. Introduction

The relationship between health and nutrition has been recognized for a long time; however, it was not until the development of high-throughput technologies that followed the sequencing of the human genome that the dynamics of this relationship could be unveiled, which was found to be influenced by the interactions between individual genetic makeup and environmental factors such as dietary components. The applications of these technologies to nutrition research led to the development of nutritional genomics which studies both the effect of an individual’s genetic makeup on the response to nutrients (nutrigenetics) and the modulation of gene expression by nutritional compounds (nutrigenomics). It is now recognized that the study of reciprocal interactions between genes and nutrients holds the key to our understanding of mechanisms underlying diseases, to the discovery of novel biomarkers of disease risk and to the development of novel food policies and therapeutic approaches adapted to the individual’s needs. As a result, there is a possibility for *personalized approach to nutrition* to emerge from this knowledge offering novel strategy for disease prevention at a population level.

A nutrient that particularly benefited from the development of nutritional genomics approaches to understand its relationship with human health and disease is the micronutrient Selenium (Se). There are three main reasons for this: (i) the risk-benefit window for Se intake is very narrow, leading to confusing results from epidemiological and intervention studies and highlighting the importance of inter-individual variations in response to Se intake; (ii) the geographical distribution of Se in the soil varies greatly with regions poor in Se such as Europe, New Zealand and some parts of China [1,2,3,4] and regions rich in Se such as the USA and Canada [5], resulting in huge differences of daily intake between populations (for example, Se daily intake in Europe is estimated at around 40  μg /day compared with around 90–134 μg /day in the USA [6]); (iii) several genetic variations in genes involved in Se metabolism were shown to interact with Se status to modulate risk for various diseases. Importantly, the application of nutritional genomics to the study of the relationship between Se and human health has not only contributed to the identification of subgroup populations at increased risk of disease but as well has provided new clues to our understanding of the metabolic function of selenoproteins, a group of 25 proteins in humans in which Se is incorporated in the form of the amino acid selenocysteine and which represent the biological actors of Se. Selenoprotein plays a key role in cellular maintenance, antioxidant defenses, endoplasmic reticulum stress, mitochondrial function and immune response [7].

This review discusses the relationship between diseases and genetic variations in selenoprotein genes with an emphasis on the function of selenoproteins and factors that governs Se homeostasis.

## 2. Selenoprotein Functions and Genetic Variants

The identification of genetic associations between single nucleotide polymorphisms (SNPs) in selenoproteins and Se-related genes has shed some light on conflictual results from epidemiological studies investigating the relationship between of Se and disease risk, and on the understanding of the role of selenoproteins in tissue function. In particular, functional studies have revealed that SNPs in Se-related genes have the potential so affect (a) Se homeostasis and selenoprotein synthesis, (b) antioxidant defenses and redox control, and (c) endoplasmic reticulum (ER) signaling and degradation of misfolded proteins (Table 1).

The table summarizes the protein name, function, subcellular and histological localization, the gene symbol and chromosomal location. Proteins are grouped according to their main function.

### 2.1. Genetic Variations in Genes Involved in Se Homeostasis

Se is incorporated in selenoprotein during their translation, in the form of the amino acid selenocysteine (Sec) a cysteine analogue in which the sulphur-containing thiol is substituted by a selenol group [8]. This mechanism is complex and involves two major regulatory elements present in selenoprotein mRNAs: a stem-loop structure called the SECIS element (Sec-Insertion Sequence) located in the 3′ untranslated region (3′UTR) of the mRNA and the recoding of a UGA codon from a premature stop codon to Sec [8]. Selenoprotein synthesis relies on the transport of dietary Se from the liver to other organs, the biosynthesis of Sec and the read-through of the UGA stop codon with a tRNA charged with Sec. In conditions of limited Se intake, these processes are tightly regulated to prioritise Se distribution to specific tissues and selenoproteins at the expense of others, in a process called the selenoprotein hierarchy [9,10]. Polymorphic variants were identified in genes involved in Sec synthesis (Sec tRNA synthase *(SEPSECS)* and selenophosphate synthetase 1 (*SEPHS1*)) and Se transport and delivery (*SEPP1*) [11,12,13]. In addition, SNPs located in the 3′UTR of *GPX4* and *SEP15* mRNAs were shown to alter the selenoprotein hierarchy [14,15,16,17]. The molecular mechanisms by which these SNPs mediate their effects on the selenoprotein hierarchy are not completely clear. Variants for rs713041 (*GPX4*) were shown to lead to transcripts with different affinity for the selenoprotein synthesis machinery and to alter to lymphocyte GPx1 and GPx4 expression *in vivo* [16] and altered selenoprotein expression and sensitivity to oxidative challenge and Se-depletion in Caco-2 cells [18]. This suggests that the location of these SNPs nearby or within the SECIS element was responsible for their effect on the selenoprotein hierarchy.

Moreover, a direct consequence of the existence of the selenoprotein hierarchy is that a SNP affecting the synthesis of one selenoprotein has the potential to affect the synthesis of other selenoproteins, with potential repercussion on the ability of an individual to response to stress [7,11,12,16].

Thus, genetic variants in genes involved in Se-metabolism have the potential to modulate the relationship between Se status and diseases by altering downstream selenoprotein synthesis. Moreover, they could represent evolutionary signatures in our genome resulting from the need during human migration to adapt to changes in Se bioavailability in different environments. Thus, not surprisingly, the association of these SNPs with disease risk was often found to be modulated by the Se status and/or the ethnicity of the population (see below) consistent with a local adaptation of populations to geographic variations of Se distribution.

**Table 1:** Se-related and selenoprotein genes linked to disease in genetic association studies.

Protein	Gene Symbol	Chromosome Location	Protein Function	Localization/Expression
***Se Metabolism and Homeostasis***				
Selenoprotein P	*SEPP1*	5q31	Plasma transporter of Sec; anti-oxidant in endothelium	Extracellular (plasma)/Liver, brain, other tissues
Selenophosphate synthetase 1	*SEPHS1*	10p14	synthesis selenophosphate from selenide and ATP	Nucleus/ubiquitous
Sec tRNA synthase	*SEPSECS*	4p15.2	conversion of O-phosphosery l-tRNA(Sec) to selenocysteiny l-tRNA(Sec)	Ubiquitous
***Redox Active Selenoproteins***				
Cellular glutathione peroxidase	*GPX1*	3p21.3	major antioxidant enzyme, detoxification of hydrogen peroxide	Cytosol/ubiquitous
Glutathione Peroxidase 3	*GPX3*	5q23	detoxification of hydrogen, redox signalling	Extracellular (plasma)/kidney, other tissues
Phospholipid Hydroperoxide Glutathione Peroxidase	*GPX4*	19p13.3	reduces phospholipid hydroperoxides, sperm maturation, redox signalling	Cytosol, membrane , mitochondria/ubiquitous, brain, testis
Thioredoxin Reductase1	*TXNRD1*	12q23.3	reduction oxidized thioredoxin and other substrates, intracellular redox control	Cytosol/ubiquitous
Thioredoxin Reductase 2	*TXNRD2*	22q11.21	reduction oxidized thioredoxin, mitochondrial redox control	Mitochondria/Liver, kidney, other tissues
***Selenoprotein Genes Involved in ER Signalling and Degradation of Misfolded Proteins***				
15KDa-selenoprotein	*SEP15*	1p31	formation of disulfide bonds, quality control of protein folding in the endoplasmic reticulum	Endoplasmic reticulum/ubiquitous
Selenoprotein S	*SELS*	15q26.3	removal of misfolded proteins from the endoplasmic reticulum lumen (ERAD pathway)	Endoplasmic reticulum/ubiquitous

### 2.2. Genetic Variations in Redox Active Selenoproteins 

Several functional genetic polymorphisms have been found in glutathione peroxidases (*GPX1*, *3*, *4*) and thioredoxin reductases (*TXNRD1–2*) gene, two major classes of redox-active enzymes involved in the control of cellular redox balance and antioxidant defenses. As described above, rs713041 in the *GPX4* gene was shown to affect the selenoprotein hierarchy by affecting the binding affinity of the selenoprotein machinery to the SECIS element in the *GPX4* mRNA [16]. In addition, a coding SNP in the *GPX1* gene rs1050450 was shown to induce a Pro to Leu amino acid change at position 198 of the amino sequence, with the Leu variant exhibiting a reduced activity [19,20]. GPx1 is one of the most important antioxidant enzymes and as a result the Leu variant has been associated with many diseases (see below). SNPs affecting the synthesis of these enzymes or their activity have the potential of weakening an individual’s capacity to respond to oxidative damage involved in the ageing process, and in most chronic diseases including cancer, cardiovascular disease, diabetes and dementia. Similarly, a number of genetic associations have now linked tagSNPs in *TXNDR1* and *2* loci with several diseases. TagSNPs are SNPs in high linkage disequilibrium (LD) with a group of other SNPs grouped in a haplotype block and can be used to capture genotype for all SNPs within the same haplotype. Importantly, LD between SNPs varies between populations, and, therefore, results from tagSNP approaches cannot necessarily be translated to other populations with different genetic backgrounds. No functional studies were carried out to determine the effect of these SNPs on *TXNRDs* functions as they probably only *tag* the existence of a functional SNP within the haplotype. However, this approach supports a role of TXNRD proteins in diseases. Finally, the identification of *GPX1*, *GPX4* and *TXNRD2* loci in several Genome Wide Association Studies (GWAS) is consistent with the crucial role of these proteins in cellular maintenance and response to oxidative stress [21,22,23,24].

### 2.3 Genetic Variations in Selenoprotein Genes Involved in ER Signalling and Degradation of Misfolded Proteins

In eukaryotic cells, the ER is responsible for intracellular Ca^2+^ homeostasis, lipid biosynthesis and protein folding and transport [25].Protein folding in the ER is highly sensitive to changes in the environment. Alterations of Ca^2+^ levels, redox state, nutrient status, protein synthesis rate or inflammatory stimuli, can disrupt protein folding leading to the accumulation of unfolded or misfolded proteins, called ER stress [25]. Accumulation of misfolded proteins is now known to play a major role not only in cancer but as well in dementia and other chronic diseases. Genetic polymorphisms in two selenoproteins located in the ER, selenoprotein S (*SELS*) and15kDa selenoprotein (*SEP15*) have now been linked to various cancers and inflammatory conditions, suggesting that these SNPs affect proper ER function.

The influence of these SNPs on disease risk and progression informs us of the key biological mechanisms implicated in the aetiology of the disease as well as of the importance of key selenoproteins in maintaining a healthy tissue. As a result of shared translation machinery and a hierarchy of distribution of Se between the different selenoproteins, the effect of one SNP on disease risk has the potential to be modulated by the individual’s Se status through gene-nutrient interactions and by various combinations of SNPs in distinct selenoprotein genes through SNP-SNP genetic interactions.

## 3. Genetic Variants in Selenoprotein Genes and Breast Cancer

Breast cancer (BC) is the most common cancer in women and a leading cause of mortality [26,27]. It is a complex and heterogeneous disease both at molecular and histological levels, with, in particular, several tumour subtypes (probably originating from distinct tumour progression pathways) associated with different clinical outcomes [28].

Because of the contribution of oxidative stress to BC pathogenesis [29], several epidemiological studies have looked at the effect of low Se intake on BC risk and an inverse correlation between BC risk and serum Se status was observed in a recent meta-analysis of 16 studies [30].

Moreover, alteration of anti-oxidant enzymes expression such as GPx1 has been observed in breast tumours. It was first reported that breast tumours present frequent loss of heterozygosity (LOH) at the *GPX1* locus associated with reduced GPx1 activity [19]. *GPX1* expression was as well silenced by aberrant hyper methylation in ~20% of primary breast cancers [31]. On the other hand, elevated GPx1 levels in breast tumours have been associated with increased mortality risk and tumour-resistance to chemotherapy [32]. This suggested that GPx1 overexpression in cancer cells was a response to oxidative damage generated by the chemotherapeutic agent, protecting cancer cells from being killed by the treatment and thus facilitating their clonal expansion [32] 

These reports indicate that factors, such as genetic variations, that modulate GPx1 activity and protein levels could affect both BC development and treatment. In addition, a polymorphism in the *GPX1* gene, resulting in various lengths for a polyalanine sequence in the N-terminal region of the protein (between 5 and 7 Ala) was found to alter subcellular localisation of GPx1 protein and response to oxidative damage in breast cancer cells [33], suggesting that GPx1 subcellular localisation plays a key regulatory role in GPx1 activity. The allele coding for the five Ala variant has been linked to increased BC risk in premenopausal women [34]. Moreover, several studies have assessed the association between genotype for *GPX1* rs1050450 (Pro198Leu) and BC risk in different populations (Table 2). It was first reported in a US population that TT carriers for rs1050450 had a higher BC risk [19]. This effect was attributed to the reduced GPx1 activity in the corresponding Leu protein variant [19]. A similar association was observed in a Danish case-control study [20] and later confirmed in an expanded analysis including 975 cases and controls matched for age and hormone replacement therapy (HRT), with Leu carriers being more prone to develop of non-ductal BC or high grade ductal BC [35]. In addition, women of TT genotype using HRT and who develop BC later in life, exhibited lower pre-diagnostic erythrocyte GPx1 (eGPx1) activity compared with controls [35]. In this study, it was shown that GPx1 protein levels increase in MCF-7 breast adenocarcinoma cells as a result of β-estradiol treatment. This observation is compatible with various reports indicating an effect of sex-hormones on GPx activity. In particular, in women blood Se levels fluctuate throughout the menstrual cycle with the highest levels coinciding with the estrogen peak [36] and in male reproductive organs, GPx4 mRNA expression is upregulated in response to estrogens [37]. In the case of BC, a dual role of sex-hormones has been proposed with increased DNA damage as a result of the generation of reactive-oxygen-species through redox cycling of the catechol estrogens, and an activation of antioxidant enzymes (MnSOD and GPx) via the NFκB pathway in animal models [38]. In the extended Danish study population, a dramatic 60% reduction in overall BC and ductal BC risks were identified in Thr carriers for rs3877899 (Ala234Thr) in *SEPP1* (Table 2), suggesting a key role of the Se transporter in the aetiology of the disease and/or breast tissue function [35]. This SNP had previously been shown to affect Se bioavailability for the synthesis of other selenoproteins [11], offering a potential mechanism by which altered Se supply to the breast tissue could contribute to BC susceptibility [35].

The table presents results from various association studies between breast cancer risk and functional and tagSNPs in selenoprotein genes. The allele or genotype associated with disease risk or progression is indicated together with the studied population and the known functional consequences of the SNP for the protein function or expression.

These observations suggest that GPx1 acts as a tumour suppressor to prevent BC development but that its anti-oxidant properties may be particularly crucial to block the development of certain subtypes of BC, and eGPx1 has the potential of becoming a biomarker for BC risk [35]. Similarly, a tumour suppressor activity of the *SEP15* gene was suggested by frequent LOH at the *SEP15* locus in breast tumours among African American women [39]. Moreover, genotype for rs5859 in *SEP15,* which had been shown to reduce the efficiency of Sec incorporation in the protein and thus its synthesis [14], was linked to BC risk in African American women but not in Caucasians [14]. In addition, no association was found between rs1050450 (*GPX1)* and BC survival, but carriers of T allele rs713041 (*GPX4)* were shown to have increased risk of mortality by BC in a British population [40].

More recently, Pellat and collaborators used a tagging SNP approach to capture genotype for all SNPs in several selenoprotein genes and assessed their influence on BC risk. They identified that SNPs in *GPX1* and *GPX3* and *SELS* genes were associated with estrogen receptor/progesterone receptor status of breast tumours. In addition, SNPs in *SEPP1* modulated BC risk whereas SNPs in *GPX4* affected survival in women of Native American ancestry [41], supporting the above observations of a role of GPx1 and SePP proteins in BC.

These observations provide some clues to explain some of the inconsistencies between epidemiological studies and between association studies carried out in different populations. The influence Se status and genetic variants in selenoproteins on BC risk is modulated by ethnicity (suggesting other genetic factors contributing to the disease), by the study design (such as the use of distinct matching criteria between the different populations or the use of tagSNPs exhibiting different LD indistinct populations), by the molecular subtypes of BC tumours and probably by additional environmental factors. Thus, the understanding of the role of SNPs in selenoproteins in relation to BC aetiology will require taking into consideration these different factors and their mutual interactions.

**Table 2:** Functional SNPs in selenoprotein genes associated with breast cancer.

Gene Symbol	SNP	Base Change	Cases/Controls	Target/Location	Functionality	Population	Association	Reference
*GPX1*	rs1050450	C > T	1038/1088	Pro198Leu	Enzymatic activity Pro > Leu	USA	none	[42]
			1229/1629			USA	none	[43]
			79/517			USA	T allele: ↑ BC risk	[19]
			399/372			Canada	none	[34]
			2293/2278			UK	none	[44]
			4371/0			UK	No association with BC survival risk	[40]
			377/377			Denmark	T allele: ↑ BC risk	[20]
			933/959			Denmark	T allele: ↑ non-ductal BC; interaction with rs3877899(SEPP1); ↓ eGPx activity	[35]
*GPX4*	rs713041	C > T	2182/2264	3'UTR, near SECIS	Sec-insertion efficiency C > T	UK	none	[44]
			4356			UK	T allele: ↑ risk of mortality by BC	[40]
			939/960			Denmark	T allele: ↓ eGPx activity	[35]
*SEPP1*	rs3877899	G > A	937/957	Ala234Thr	Plasma SePP isoforms, Se bioavailability	Denmark	AA: ↓ BC and ductal BC risk	[35]
*SEPP1*	rs7579	G > A	937/957	3'UTR	Plasma SePP isoforms, Se bioavailability	Denmark	none	[35]

## 4. Genetic Variants in Selenoprotein Genes and Prostate Cancer

Prostate cancer (PCA) is the second most common cancers among men worldwide, and is a leading cause of cancer death. Its incidence is increasing in nearly all countries and is much higher in Western countries [45]. The disease is caused by a combination of diet, lifestyle, environmental and genetic factors [46]. Results from the NPC trial have sparked great interest for Se, as the randomised clinical trial (RCT) revealed a dramatic 65% reduction of PCA in men supplemented with Se [47]. The effect was particularly evident for individuals with baseline Se status < 106 μg/L [48]. On the other hand, the Selenium and Vitamin E Cancer Prevention Trial (SELECT), showed no benefit of Se alone but identified a significant 17% increased risk of developing PCA in men supplemented with Vitamin E alone compared with placebo, and a non-significant increase in PCA cases in men supplemented with combined Se and Vitamin E [49,50]. Furthermore, Se supplementation of participants who had a high baseline Se status was shown to increase risk of high-grade PCA [51]. This result contrasts with previous findings from the prospective Netherlands Cohort Study indicating an inverse association between toenails Se and advanced PCA risk in a European population with much lower Se status [52] but support results from other observational studies [53,54].

Recent meta-analyses of observational studies suggest that high Se concentrations in plasma, serum, or toenails may be linked to reduced PCA risk [55,56,57,58]. However, they identified a complex U-shaped relationship between Se status and PCA risk, suggesting the existence of a Se status threshold below which raising Se intake could protect against PCA, but above which supplemental Se provides no additional benefit, and can even be harmful [59]. This observation offers a possible interpretation for the discrepancy between the SELECT study and the other studies, as SELECT participants had a much higher baseline Se status, ranging from 122–152 μg/L (mean 136 μg/L) compared with the NPC trial or with Se status observed in European populations [52,58].

Additional evidence coming from genetic association studies support an interaction between Se status, genetic variants in selenoprotein genes and risks of PCA or aggressive PCA (Table 3). In particular, two functional SNPs in the *SEPP1* gene, previously shown to alter Se bioavailability, affect PCA risk in several European populations [60,61]. In two distinct European studies, homozygous AA for rs7579 (*SEPP1*) were found to have an increased risk for PCA (OR, 1.72 [0.99–2.98]; *P* = 0.05) [60] and for advanced PCA [52]. Moreover, a significant interaction between rs3877899 (*SEPP1*) and rs4880 (a SNP in *SOD2* gene, coding for the manganese superoxide dismutase) was found to modulate PCA/aggressive PCA risks in smokers [61]. In addition, three genetic variations in *GPX1* were shown to modulate PCA risk in different European populations, with genotype for rs1050450 found to modify the relationship between serum Se concentration and disease risk, with men carriers of the T allele having a reduced risk of PCA (odds ratio (OR) = 0.87 [0.76–0.99]; *P* = 0.04, *P*_interaction_ = 0.03) or high-grade PCA (OR = 0.64 [0.49–0.83]; *P* = 0.001, *P*_interaction_ < 0.001) per 10 μg/L increase in serum Se concentration [60] and genotype for rs17650792 and rs1800668 linked to advanced PCA risk [52].

**Table 3:** Functional SNPs in selenoprotein genes associated with prostate cancer.

Gene Symbol	SNP	Base Change	Cases/Controls	Target/Location	Functionality	Population	Association	Reference
*GPX1*	rs1050450	C > T	745/0	Pro198Leu	Enzymatic activity Pro > Leu	USA	none	[62]
			500/1391			USA	none	[63]
			247/487			Germany	T allele: ↓ PCA risk with ↑ serum Se levels	[60]
			82/123			Macedonia	T allele: ↓ PCA risk	[64]
			262/435			New Zealand	T allele: ↑PCA risk	[65]
*GPX1*	rs1800668	C > T	951/25408	tagSNP/promoter	tagSNP/high LD with rs1050450	Netherlands	TT: ↓advanced (stage III/IV) PCA risk	[52]
*GPX1*	rs17650792	A > G	952/25426	tagSNP/promoter	unknown	Netherlands	GG: ↑advanced (stage III/IV) PCA risk	[52]
*GPX4*	rs713041	C > T	739/0	3'UTR, near SECIS	Sec-insertion efficiency C > T	USA	none	[62]
			245/490			Germany	none	[60]
			260/439			New Zealand	none	[65]
*SEPP1*	rs3877899	G > A	2643/1570	Ala234Thr	Plasma SePP isoforms, Se bioavailability	Sweden	none	[61]
			248/492			Germany	none	[60]
			951/25409			Netherlands	genotype interacts with Se status to ↓advanced PCA risk	[52]
			259/436			New Zealand	none	[65]
*SEPP1*	rs7579	G > A	248/492	3'UTR	Plasma SePP isoforms, Se bioavailability	Germany	AA :↑ PCA risk; interaction with plasma [SePP]	[60]
			951/25408			Netherlands	A allele: ↓advanced (stage IV) PCA risk; genotype interacts with Se status to ↓advanced PCA risks	[52]
*SEPP1*	rs13168440	T > C		tagSNP	unknown	USA	C allele: interacts with Plasma Se to ↓PCA risk	[66]
*SEP15*	rs5859	G > A	1195/1186	3'UTR	Sec-insertion efficiency	USA	none	[67]
			248/492			Germany	AA :↓ GPX3 activity	[60]
*SEP15*	rs5845	G > A or C > T	259/436	3'UTR	Sec-insertion efficiency	New Zealand	AA ↑ PCA risk	[65]
*SEP15*	rs561104	G > A	1195/1186	tagSNP	unknown	USA	AA: ↑risk of mortality by PCA	[67]
SELK	rs9880056	T > C	248/492	tagSNP	unknown	Germany	C allele: interacts with serum SePP and serum Se to ↓ risk advanced and high grade PCA	[68]
*TXNRD1*	rs7310505	C > A	248/492	tagSNP	unknown	Germany	CC: interacts with serum SePP and serum Se activity to ↑ risk of advanced PCA	[68]
*TXNRD2*	rs9605030	C > T	248/492	TagSNP	unknown	Germany	T allele: interact with serum Se concentration to ↑ high grade PCA risk	[68]
*TXNRD2*	rs9605031	C > T	248/492	TagSNP	unknown	Germany	T allele: interact with serum Se concentration to ↓ high grade PCA risk	[68]

The table presents results from various association studies between prostate cancer risk and functional and tagSNPs in selenoprotein genes. The allele or genotype associated with disease risk or progression is indicated together with the studied population and the known functional consequences of the SNP on the protein function or expression.

In a nested case-control study within the Physicians Health Study in the US, Penney and collaborators identified that three variants in *SEP15 (*rs479341, rs1407131 and rs561104) significantly associated with PCA-related mortality. In addition, an inverse relationship between high levels of Se and PCA mortality was observed among men who did not have the increased risk genotype for rs561104, revealing the PCA mortality was modulated by the interaction between rs561104 and Se [67]. Using tagSNPs to capture all SNP at the *SEPP1* locus, the authors ssubsequently identified two genetic variations in the *SEPP1* gene that influence prostate cancer incidence [66]. In this study, a significant interaction between rs13168440 (*SEPP1*) and plasma Se levels was observed, with a reduced risk of PCA for individuals with high Se status carrying the minor C allele but not for TT men. On the other hand, the T allele was associated with an increased mortality in smokers or obese individuals [66]. Supporting this effect of SNPs on PCA survival, tagSNPs in *TXNRD1* (rs10778322, rs1128446, rs4964785) and *GPX4* (rs2074452) were shown to be modestly associated with risk of mortality by prostate cancer [69].

Finally, in a case-control study nested within the EPIC-Heidelberg population, a pathway-wide analysis, using 384 tagSNPs, was developed to simultaneously genotype all SNPs in 72 genes. This alternative approach revealed that risk for high-grade or advanced stage of PCA was significantly modified by interactions between plasma markers of Se status (plasma Se, SePP and GPx3 activity) with SNPs in *SELK* (rs9880056), *TXNRD1* (rs7310505) and *TXNRD2* (rs9605030 and rs9605031) genes [68]. The link between SNPs in *TXNRD1-2* and *SEP15* and prostate cancer incidence was recently supported by evidence that an association between genetic variants in these genes and risk of high-grade prostate cancer and prostate cancer recurrence [70]. The influence of genetic variants in selenoprotein genes on disease stage was further supported by observations that rs7579 (*SEPP1*) and rs17650792 and rs1800668 (*GPX1*) modulate risk for advanced PCA stages [52].

## 5. Genetic Variants in Selenoprotein Genes and Colorectal Cancer

Colorectal cancer (CRC) is the second most frequent cause of death by cancer in Europe and the third most common cancer worldwide [71]. Several risk factors for CRC have been identified including the interaction between genetic factors and lifestyle factors such as diet [72]. Both mechanistic and epidemiological evidence has suggested a beneficial role of Se in CRC prevention [73]. Strong interests for Se were motivated by results from the National Prevention of Cancer (NPC) trial, revealing a ~60% reduction in CRC cases in individuals supplemented with Se-enriched yeast (200 μg/day) compared with placebo for 4.5 years and then followed up for a further 6.5 years [47,74]. Supplementation was most effective in participants with low plasma Se (<106 μg/L) before supplementation. Unfortunately, no beneficial effects of Se supplementation were observed in in the large Se and Vitamin E Cancer prevention Trial (SELECT) [50]. As for PCA, the discrepancy between the two studies was attributed to the use of different source of Se in the supplement and to the fact participants from the SELECT study had a much higher plasma Se status at baseline compared with people who took part in the NPC study. Supporting this interpretation, recent data from an EPIC case-control study indicated that low plasma Se status (~80 μg/L), as observed in most European countries, was associated with increased CRC incidence in women [75]. Thus, increased Se intake may contribute to lower CRC risk in people with a plasma Se (<100 μg/L) but not in individuals with high Se intake such as the ones observed in US and Canadian populations [75].

In addition, genetic association studies carried out in different populations have provided evidence that the effect of Se status on CRC or colorectal adenoma risks could be modulated by genetic variations in selenoprotein genes (Table 4). In a case-control study carried out on a US population, the risk of advanced distal colorectal adenoma was associated with various genetic variants in *SEPP1* and *TXNRD1* genes but not with SNPS in *GPX1*, *GPX2*, *GPX3*, and *GPX4* [76].

CRC risk was shown to be modulated by genotype for rs713041 in the *GPX4* gene, a SNP affecting Sec incorporation and selenoprotein hierarchy [16,17]. In a small Scottish study (~300 cases, 189 controls), CC carriers exhibited a significantly lower erythrocyte GPx1 activity (eGPX) and an increased CRC risk [77].On the contrary, in a Czech population (832 cases, 705 controls), the T allele was associated with greater CRC risk [78] and no association was observed in a Korean population (827 cases, 727 controls) [79]. No data on Se and Se-biomarker status were available for the Korean and Czech populations, suggesting that differences in Se status between the distinct populations could explain some of the inconsistency between the studies. In addition, genetic interactions between rs713041 and SNPs in *SEPP1*, *SOD2* and *TXNRD2* genes were found to further modified the disease risk in the Czech population [78], suggesting a complex polygenic contribution to the disease aetiology. Since MnSOD and TXNRD2 proteins play a key role in the control of the mitochondrial redox status [78] and since *GPX4* knock-down in Caco-2 gut epithelial cells results in the disruption of mitochondrial function [80], this suggests that of these SNPs may impact disease risk by affecting mitochondrial function and may have different consequences depending on the environmental/lifestyle factors associated with CRC in these distinct populations. Moreover, the genetic interaction between rs3877899 (*SEPP1*) and rs713041 suggests that the reduced binding affinity for the selenoprotein synthesis machinery carried by the T allele for rs713041 could be amplified in carriers of a variant in *SEPP1* associated with reduced Se bioavailability [11,12,16]. As a result of the genotypic combination, *GPX4* expression will be decreased and the availability of the synthesis machinery for the translation of selenoproteins lower in the hierarchy will be increased [16]. The overall change in the proportion of selenoproteins being translated could, in turn, modulate the capacity of an individual to respond to specific stressors and CRC risk. From these studies, it remains unclear whether the influence of rs713041 on GPx4 expression or on the synthesis of other selenoproteins is responsible for its association with CRC risk, but it becomes apparent that multiple factors can affect the impact of the SNP on the disease aetiology.

**Table 4:** Functional SNPs in selenoprotein genes associated with colorectal cancer.

Gene Symbol	SNP	Base Change	Cases/Controls	Target/Location	Functionality	Population	Association	Reference
*GPX1*	rs1050450	C > T	656/743	Pro198Leu	Enzymatic activity Pro > Leu	USA	no association with advanced distal colorectal adenoma	[76]
			981/397			Norway	none	[81]
			375/779			Denmark	none	[82]
			832/705			Czech Republic	no association alone, but genetic interaction with rs37413471 (*SELS*)	[78]
			827/733			Korea	none	[79]
*GPX4*	rs713041	C > T	745/758	3′UTR, near SECIS	Sec-insertion efficiency C > T	USA	no association with advanced distal colorectal adenoma	[76]
			252/187			UK	TT:↓ CRC risk	[77]
			832/705			Czech Republic	CT: ↑ CRC risk; interaction with rs4880 (*SOD2*), rs9605031 (*TXNRD2*) and rs3877899 (*SEPP1*)	[78]
			827/733			Korea	none	[79]
*SEPP1*	rs3877899	G > A	193/127	Ala234Thr	Plasma SePP isoforms, Se bioavailability	Germany	none	[83]
			832/705			Czech Republic	No association alone, but interaction with rs5859(*SEP15*) and with rs713041 (*GPX4*)	[78]
			827/733			Korea	none	[79]
*SEPP1*	rs7579	G > A	832/705	3′UTR	Plasma SePP isoforms, Se bioavailability	Czech Republic	AA:↑ CRC risk ge, interaction with rs5859 (*SEP15*)	[78]
			827/733			Korea	none	[79]
*SEPP1*	Promoter (−4166), Exon 5 (rs3877899, rs6413428), 3'UTR (rs12055266, rs2972994, rs3797310)		772/777			USA	Global *SEPP1* variants association with advanced distal colorectal adenoma	[76]
*SEP15*	rs5859		832/705	3'UTR, SECIS	Sec-insertion efficiency	Czech Republic	no association alone, but interaction with rs3877899, rs7579, rs3797310, rs12055266 in *SEPP1*	[78]
			827/733			Korea	A allele:↑ CRC risk	[79]
*SEP15*	rs5845		827/733	3′UTR	Sec-insertion efficiency	Korea	none	[79]
	rs35009941		772/777	C > G		USA	G allele:↓ CRC, alone and in association with rs34195484, rs4077561, rs1128446, rs5018287, rs6539137,rs10778322 and rs35776976 in *TXNRD1*	[76]
*TXNRD1*	rs35009941		772/777	C > G		USA	G allele:↓ CRC, alone and in association with rs34195484, rs4077561, rs1128446, rs5018287, rs6539137, rs10778322 and rs35776976 in *TXNRD1*	[76]

The table presents results from various association studies between colorectal cancer risk and functional and tagSNPs in selenoprotein genes. The allele or genotype associated with disease risk or progression is indicated together with the studied population and the known functional consequences of the SNP on the protein function or expression.

In addition to rs713041, two other SNPs contributed to CRC risk in the Czech population: rs34713741 in the promoter region of *SELS* and rs7579 inducing a G/A change in the 3′UTR of the *SEPP1* mRNA [78]. The increased CRC risk of CRC in carriers of the T allele for rs34713741 (odds ratio: 1.68) [78] was replicated in a very distinct Korean population (odds ratio: 2.25) [79]. Rs34713741 had been previously linked to changes in plasma cytokine levels (IL1 and TNF-alpha) [84] and gastric cancer risk [85]. SelS is known for its key role in healthy colorectal function and its involvement in the ERAD (endoplasmic reticulum-associated protein degradation) and inflammation pathways, indicating that genotype for rs3471374 could contribute to CRC by affecting these functions. Moreover, rs5859 (*SEP15*) induces a base change in the SECIS element of *SEP15* mRNA resulting in an alteration Sec insertion, via a mechanism comparable to rs713041 in *GPX4*. Sep15 like SelS is involved in in the control of protein folding in the ER and genetic interactions were identified between rs7579 and rs3877899 in the *SEPP1* gene and rs5859 in relation to CRC risk [78]. Similarly to rs713041 in *GPX4*, these interactions could result in an altered SEP15 expression in condition of low Se bioavailability, and consequently could impact on protein folding regulation [78].

The role of SePP in CRC carcinogenesis was further highlighted by the observation of a reduced expression of the protein in tumour samples compared with normal mucosa and the genomic instability of a polymorphic (TC)5/(TC)3 repeats in the *SEPP1* promoter in CRC cases [83,86] and the identification of a polymorphism at *SEPP1* promoter region (TC)5/(TC)3 repeats and colon cancer risk [83]. Moreover, in a US population, genetic variants in *TNXRD2*, *Selenoprotein N*, and *Selenoprotein X* genes were associated with survival after diagnosis with rectal cancer whereas SNPs in *Selenoprotein N* and *Selenoprotein X* were linked to colon cancer mortality and SNPS in *TNXRD1-2* were shown to interact with both aspirin and cigarette smoking to alter colon and rectal cancer risk [87].

These studies emphasise the role of selenoproteins SePP, GPx4, TXNRD2, SelS and SeP15 in CRC and stress the importance of redox control and control of protein folding in colorectal function. Moreover, genetic interactions between variants in *SEPP1,* affecting Se bioavailability, and SNPs in *SEP15* and *GPX4*, affecting Sec insertion, underline the importance of the selenoprotein hierarchy in this tissue and the need for adequate Se supply to support selenoprotein synthesis to maintain a healthy colorectum.

## 6. Genetic Variants in Selenoprotein Genes and Other Conditions

Genetic variants in selenoproteins have been involved in other cancers and diseases. In particular, rs1050450 (*GPX1*) has been linked to other cancers such as lung [88,89,90,91], bladder [92,93] and laryngeal [90] cancers (Table 5). Furthermore, a combination of two SNPs in *GPX1* in high linkage disequilibrium, rs1050450 and rs18006688, was found to significantly modify the association between lead exposure and glioblastoma [94], suggesting that the reduced GPx1 activity of this variant could weaken protection against oxidative damage generated by lead. In a meta-analysis, investigating the contribution of genotype for rs1050450 to tumour risk in different populations from 31 case-control studies, revealed an increased cancer risk (OR = 1.12, [1.02–1.23]) for carriers of the Leu variant [95]. Furthermore, rs1050450 was associated with metabolic syndrome and obesity [96,97], in relatively small studies and carriers of the T allele had at significant higher risk of developing Kashin-Beck disease (KBD) in a Chinese Han population deficient in Se [98]. This was associated with a reduced blood GPx activity and *GPX1* and NF-kappa-B p65 mRNA levels in whole blood and cartilage tissue samples of KBD patients [98]. Similarly, *GPX4* mRNA level was dramatically lower in the blood of KBD patients and a significant haplotype A-T from the combination of two genetically linked SNPS in *GPX4* (rs713041 and rs4807542) was shown to protect against KBD [99].

The table presents results from various association studies between disease risk and functional and tagSNPs in selenoprotein genes. The allele or genotype associated with disease risk or progression is indicated together with the studied population and the known functional consequences of the SNP on the protein function or expression.

In a Caucasian population from New Zealand, where the soil is poor in Se and Se intake is low [100], rs7901303 *and* rs17529609 *in* selenophosphate synthetase 1 (*SEPHS1*) and rs1553153 in Sec tRNA synthase *(SEPSECS)* were significantly associated with Crohn’s Disease [13]. Both proteins play a key role in selenoprotein synthesis, via their implication on the synthesis of selenocysteine and SectRNA. The authors found that Se status was lower in Crohn’s Disease patients compared with controls [13]. Thus, more research to study the effects of these genetic variants on selenoprotein synthesis and Se homeostasis could help to understand the relationship between Crohn’s Disease and Se and potential consequences for CRC risk.

Moreover, GWAS studies revealed that *GPX1* and *GPX4* loci were associated with Crohn’s disease and inflammatory bowel disease [21,22] whereas *TXNRD2* locus was associated with susceptibility to *staphylococcus aureus* infection [24] and glaucoma [23].

Interestingly, several SNPS is the selenoprotein S gene were shown to affect risk of several chronic diseases. In particular, rs28665122 in the promoter of *SELS*, known to influence pro-inflammatory cytokines levels [84], contributes to Hashimoto’s thyroiditis susceptibility, with carriers of the A allele having a highly significant increased risk of developing Hashimoto’s thyroiditis [101]. In addition, this A allele was also shown to be significant risk factor for pre-eclampsia [102]. On the contrary, in a Spanish cohort no association of rs28665122 and five other SNPs in *SELS* was observed with type 1 diabetes, rheumatoid arthritis and the inflammatory bowel diseases, Crohn’s disease and ulcerative colitis [103]. Moreover, several tagSNPs in the *SELS* gene were shown to affect markers of cardiovascular disease risk in European American families enriched for type 2 diabetes [104] and epistasis between interleukin 1 and a SNP in selenoprotein S promoter was shown to modulate susceptibility to rheumatoid arthritis [105].

Finally, the coding SNP Thr92Ala (rs225014) in type 2 deiodinase (*IDI2*) gene has been linked with type 2 diabetes [106,107,108] and an association of four SNPs in the *SEPP1* gene (2 coding SNPs: rs28919926; and rs146125471; one intronic SNP: rs16872779 and rs7579 in the 3′UTR) with fasting insulin and first phase insulin response was replicated in Hispanic cohorts [109]. These results support the observations of the role of selenoprotein P in the regulation of glucose metabolism and insulin sensitivity [110,111].

**Table 5:** Functional SNPs in selenoprotein genes associated with other diseases.

Gene Symbol	SNP	Base Change	Cases/Controls	Target/Location	Functionality	Population	Association	Reference
***LUNG CANCER***								
*GPX1*	rs1050450	C > T	237/234	Pro198Leu	Enzymatic activity Pro > Leu	USA	Among old smokers, CC :↑ lung cancer	[88]
			315/313			Finland / men	T allele: ↑ risk	[89]
			95/176			Poland	T allele: ↓ risk	[90]
			432/798			Denmark	T allele: ↓ risk	[91]
			186/207			Germany	T allele: ↓ risk	[112]
*GPX4*	rs713041	C > T	95/176	3′UTR, near SECIS	Sec-insertion efficiency C > T	Poland	T allele: ↓ risk	[90]
*SEP15*	rs5859		325/287	3′UTR, SECIS	Sec-insertion efficiency	Poland	A allele: ↑risk in individuals with low Se status	[113]
*SEP15*	rs5845		325/287	3′UTR	Sec-insertion efficiency	Poland	none	[90]
***LARYNGEAL CANCER***								
*GPX1*	rs1050450	C > T	111/213	Pro198Leu	Enzymatic activity Pro > Leu	Poland	T allele: ↓ risk	[90]
*GPX4*	rs713041	C > T	325/287	3′UTR, near SECIS	Sec-insertion efficiency C > T	Poland	T allele: ↓ risk	[90]
*SEP15*	rs5845		325/287	3′UTR	Sec-insertion efficiency	Poland	none	[90]
***BLADDER CANCER***								
*GPX1*	rs1050450	C > T	224/0	Pro198Leu	Enzymatic activity Pro > Leu	USA	T allele: ↑ bladder cancer recurrence risk	[93]
			213/209			Japan	T allele: ↑ risk	[92]
***CARDIOVASCULAR DISEASE ***								
*GPX1*	rs1050450	C > T	184/0	Pro198Leu	Enzymatic activity Pro > Leu	Japan/diabetic	T allele: ↑ CVD risk in diabetic patients and ↑ intima-media thickness	[114]
*SELS*	rs28665122, rs4965814, rs28628459, rs7178239			tagSNPs		European Americans/ diabetic	Associated with measures of vascular calcification in European American familiesenriched for type 2 diabetes	[104]
***KASHIN-BECK***								
*GPX1*	rs1050450	C > T	638/324	Pro198Leu	Enzymatic activity Pro > Leu	China	none	[115]
						China Han	T allele: ↑ KBD risk	[98]
*GPX4*	rs713041	C > T	219/194	3′UTR, near SECIS	Sec-insertion efficiency C > T	China	none; ↓ GPX4 mRNA expression in Kashin-Beck patients	[99]
	haplotype rs713041 -rs4807542		219/194			China Han	haplotype A-T: ↓ KBD risk	[99]
								
*SEPP1*	rs3877899	G > A	167/166	Ala234Thr	Plasma SePP isoforms, Se bioavailability	China	none	[116]
***CROHN’S DISEASE***								
*SEPHS1*	rs7901303	G > T	351/853	tagSNP		New Zealand-Caucasians	SNP-Serum Se interaction affecting Crohn’s disease risk	[13]
	rs17529609	A > G	351/853	tagSNP		New Zealand-Caucasians	SNP-Serum Se interaction affecting Crohn’s disease risk	[13]
*SEPSECS*	rs1553153	G > A	351/853	tagSNP		New Zealand-Caucasians	SNP-Serum Se interaction affecting Crohn’s disease risk	[13]
***TYPE 2 DIABETES***								
*IDI2*	rs225014		721	Thr92Ala		Brazil	Ala variant: less active, associated with type 2 diabetes, interaction with PPARγ2 Pro12Ala	[106–108]
*SEPP1*	rs28919926, rs146125471, rs16872779, rs7579		2446			Hispanics, European American, African American	Associated with fasting insulin and first phase insulin response	[109]

## 7. Conclusions and Perspectives

Mechanistic and epidemiological studies have shown that Se and selenoproteins play a key role in biological pathways, such as cellular maintenance, protein folding and oxidative stress response, known to be affected in multifactorial chronic diseases [7]. As a result, alteration of selenoprotein synthesis or activity has been linked to development of chronic diseases such as cancer [59]. In addition, the application of Nutritional Genomics approaches to Se biology has highlighted that genetic variants in selenoprotein and Se-related genes affect the risk for multifactorial diseases, uncovering potential roles for particular selenoproteins in specific disease or tissue function. These associations could reflect the influence of these SNPs on the selenoprotein hierarchy, Se and Sec bioavailability and response to stresses. Additionally, SNPs in selenoprotein genes were found to interact with biological markers of Se status (including plasma Se and SePP levels and eGPx activity) and genetic variants in other selenoprotein genes or associated molecular pathways (such as SNPs in *SOD2* gene). This observation reveals an extra level of complexity when considering the relationship between Se and health, as the effects of a genetic variant on disease risk not only depends on the function of the corresponding protein but as well on the biological pathway it is involved in (and the presence of SNPs altering this pathway) and on the hierarchical distribution of Sec between selenoproteins and Se bioavailability. In addition, several studies noted that these interactions were observed in certain ethnic groups or populations with different Se supply or genetic background. This suggests that, during evolution, the fixation of these SNPs in sub-group populations reflected probably the need for an adaptation to both changes in environmental Se levels and exposure to specific environmental stressors. Thus, a real appreciation of the effect of Se-gene interaction on cell responses and disease prevention will require, in the future, taking into account not only the effect of Se supply on multiple selenoproteins; it will also require considering the impact of changes in selenoprotein synthesis and activity on downstream molecular pathways and on the response to environmental stresses, as well as the contribution of these variants to disease risk within the genetic background of an individual. 

Another lesson from genetic epidemiological studies is that the role of selenoproteins and the consequences for downstream molecular targets may differ between tissues. These observations uncovered potential new clues to understanding the role of specific selenoproteins in certain tissues and molecular pathways and their implication in mechanisms underpinning diseases. The study of these mechanisms, using a functional genomics approach, has the potential to contribute to the identification of early disease susceptibility markers and to the design of novel disease prevention strategies, based on *personalised dietary recommendations* of Se to individuals, depending on their genetic makeup, Se status and other risk factors.

Finally, genetic associations provided strong evidence of a link between genetic variants in selenoproteins genes and risk for various chronic diseases but failed to demonstrate how these variants formally affect disease aetiology. Thus, in the future, more studies need to be designed to not only replicate these associations in multiple and larger cohorts but as well take into account measures of Se status, gene expression and functional approaches to formally establish and quantify the contribution of combined and individual SNPs to the disease risk. To clarify the effect of the SNPs, the design of these studies would have to include stratifications according to Se levels, genotype, disease stages and response to treatment.
